# Neurodegeneration and Sensorimotor Function

**DOI:** 10.3390/brainsci10110808

**Published:** 2020-11-01

**Authors:** Matteo Bologna, Giulia Paparella

**Affiliations:** 1 Department of Human Neurosciences, Sapienza University of Rome, 00,185 Rome, Italy; 2 IRCCS Neuromed Pozzilli (IS), 86077 Pozzilli, Italy; giulia.paparella@uniroma1.it

## Abstract

Sensorimotor integration is an essential function for both motor control and learning. Over recent decades, a growing body of evidence has emerged in support of the role of altered sensorimotor integration in the pathophysiology of various neurological conditions and movement disorders, particularly bradykinesia, tremor, and dystonia. However, the various causes and mechanisms underlying altered sensorimotor integration in movement disorders are still not entirely understood. The lack of complete insight into the pathophysiological role of altered sensorimotor integration in movement disorders is certainly due to the heterogeneity of movement disorders as well as to the variable occurrence of neurodegenerative phenomena, even in idiopathic movement disorders, which contribute to pathophysiology in a complex and often not easily interpretable way. Clarifying the possible relationship between neurodegenerative phenomena and sensorimotor deficits in movement disorders and other neurological conditions may guide the development of a more detailed disease prognosis and lead, perhaps, to the implementation of novel and individualized therapeutic interventions.

Sensorimotor integration is an essential function for both motor control and learning and is subserved at various levels of the central nervous system [1]. At the cortical level, sensorimotor integration mechanisms mediate the so-called long latency or transcortical reflexes involved in the regulation of muscle tone [2], as well as the plasticity mechanisms involved in associative learning [3]. In humans, the short-latency afferent inhibition (SAI) protocol represents an in-vivo marker of sensorimotor integration [4,5]. Compelling evidence indicates that SAI is modulated in association with motor control and learning in humans [6]. The basal ganglia represent an additional neuroanatomical site involved in sensorimotor integration [7,8]. Furthermore, at the subcortical level, sensorimotor integration is one of the main physiological prerogatives of cerebellar systems [9]. At the cerebellar level, sensorimotor integration is based on feedback or feed-forward systems that are involved in various forms of motor control and learning, including sensorimotor learning, a long process connected to motor practice [10]. Finally, at the level of the brainstem and spinal cord circuits, sensorimotor integration is mainly involved in the regulation of various reflexes, including those involved in the automatic regulation of muscle tone [11,12]. Over recent decades, a large body of evidence has emerged in support of the role of altered sensorimotor integration in the pathophysiology of various neurological conditions and movement disorders [13,14,15]. It is known that altered sensorimotor integration results in altered kinematic parameters of voluntary movement and therefore in the generation of bradykinesia [16]. Accordingly, SAI has been demonstrated to be altered in Parkinson’s disease and in other neurodegenerative conditions [16,17,18]. It is also known that altered sensorimotor integration may contribute to triggering oscillatory activity, i.e., synchronous activity, in various areas that can be translated into repetitive and alternating activation of antagonistic muscle groups and the subsequent appearance of tremor [19,20]. In addition, altered sensorimotor integration can be implicated in maladaptive plasticity phenomena or other dysfunctional mechanisms, which can lead to co-contraction of antagonistic muscle groups involved in the genesis of dystonic movements [3,21]. Thus, the main question to be answered regards the identification of the mechanisms leading to altered sensorimotor integration in movement disorders, which is no easy task considering that movement disorders are a group of heterogeneous conditions, both from a phenomenological and pathophysiological point of view. It is therefore conceivable that the causes and mechanisms of altered sensorimotor integration vary greatly in each specific case. Moreover, it is also likely that the role of altered sensorimotor integration differs depending on the pathophysiological context in which it occurs.

Recently, it has been demonstrated that traumatic brain injuries trigger a molecular cascade involving oxidative stress, Ca2+ influx, and specific growth factors that causes neuroinflammation and neurodegeneration and sensorimotor deficits in animal models [22]. Moreover, in humans, it is well known that structural lesions at various levels of the central nervous system, particularly at the levels of the basal ganglia and cerebellum due to an expansive lesion or trauma can alter sensorimotor integration processes, thus leading to the appearance of different movement disorders [23,24]. In addition, the neurodegenerative processes that characterize some of the major movement disorders in humans, particularly Parkinson’s disease and Huntington’s chorea, can affect various brain areas other than the basal ganglia, thus contributing to altered sensorimotor integration processes in a complex and often not easily interpretable way. Cerebellar neurodegenerative changes have recently been described both in Parkinson’s disease and Huntington’s chorea [25,26]. These findings are of interest if we consider that both Parkinson’s disease and Huntington’s chorea have traditionally been considered expressions of basal ganglia dysfunction and not cerebellar dysfunction. Suffice to say that the cerebellum is not contemplated in the classic Braak model in Parkinson’s disease [27]. The finding of anatomopathological involvement of the cerebellum in Parkinson’s disease and Huntington’s chorea requires a reappraisal of pathophysiological models and the role of altered sensorimotor integration in these pathological conditions. Once interpreted as a dysfunction of the basal ganglia alone, Parkinson’s disease and other movement disorders are now better explained on the basis of a more extensive network dysfunction that includes the cerebellum [7,8,16,28,29]. We must also consider other movement disorders, namely the so-called idiopathic disorders, particularly essential tremor and idiopathic dystonia, where, by definition, there is no obvious structural alteration of the central nervous system. In these cases, it is not entirely clear what mechanisms underlie altered sensorimotor integration [21]. One of the most accredited hypotheses is that altered sensorimotor integration in the absence of obvious structural alterations of the cerebral parenchyma reflects the dysfunction of neuronal electrophysiological mechanisms on a genetic basis [28,30]. As previously mentioned, it is possible that several electrophysiological alterations may mediate maladaptive cortical plasticity or excitability changes of inhibitory circuits [3,30]. However, it must be noted that even idiopathic movement disorders can be characterized by neurodegenerative aspects to some extent. For example, studies have demonstrated cerebellar neurodegenerative alterations in essential tremor from early stages of the disease [31]. Similar evidence, although less compelling, also exists for dystonia [32].

The possible relationship between neurodegenerative and pathophysiological aspects, especially in regard to sensorimotor deficits, is still under-investigated and poorly understood. There is evidence that neurodegenerative processes alter the inhibitory control and plasticity mechanisms essential for proper sensorimotor integration [31,33]. However, in order to better interpret the relationship between neurodegenerative phenomena and altered sensorimotor integration, it is necessary to more precisely delineate the factors influencing the type and extent of neurodegenerative involvement of specific brain areas. For example, it has been hypothesized that neurodegenerative brain phenomena can propagate in the brain following certain specific anatomical and functional connectivity pathways [34]. In this regard, a certain connectivity pattern between brain areas could also influence genetic expression in interconnected brain areas, which could make them susceptible, to a similar extent, to accumulated neurodegenerative phenomena [35]. Another non-mutually exclusive hypothesis is that neurodegenerative phenomena can accumulate in certain hyperactive nodes (epicenters) of a given dysfunctional network [35]. The general message that we can draw from these studies is that a better pathophysiological understanding of a given movement disorder could perhaps guide us in predicting the evolution of neurodegenerative aspects, formulating a more detailed disease prognosis, and implementing individualized therapeutic interventions.
